# Improved Current Sensor for Water Diffusion Testing of Composite Insulators

**DOI:** 10.3390/s19040778

**Published:** 2019-02-14

**Authors:** Zhonghao Zhang, Fanghui Yin, Liming Wang, Hongwei Mei

**Affiliations:** Department of Electrical Engineering, Graduate School at Shenzhen, Tsinghua University, Shenzhen 518055, China; zzhzaihq@163.com (Z.Z.); AndyYinEE@gmail.com (F.Y.); mei.hongwei@sz.tsinghua.edu.cn (H.M.)

**Keywords:** water diffusion, electrode, current sensor, interface, composite insulator

## Abstract

An improved current sensor aimed at measuring currents of different parts in composite insulator samples was proposed. Conventional current sensors used in water diffusion tests aim to examine the performance of composite insulators, however, it is difficult for the conventional current sensors to locate the defects. Thus, we designed a new electrode structure to measure the currents of different components in short samples of composite insulators. Based on a finite analysis method, the influence of relative permittivity and conductivity on the current was analyzed. New samples with different interfaces and samples after operation were tested using the new and conventional current sensors. The performance of a certain part in short samples can be diagnosed by analyzing the current and phase information extracted from the test results. By comparing the test results of new and traditional current sensors, it was proved that the new electrode structure is more effective in locating the defects of insulators.

## 1. Introduction

Composite insulators, composed of a silicone rubber sheath and a fiber reinforced plastic (FRP) core rod, are widely used in transmission lines all over the world [1]. Millions of composite insulators are installed in the power grid in China, so the performance of composite insulators is of great importance to the correct operation of the power grid. In recent years, abnormal heating and fracture accidents of composite insulators have occasionally occurred in China’s power grid [2,3]. These accidents caused great economic losses and attracted the attention of researchers. Current research indicates that the poor interface performance of composite insulators is the main reason for this type of failure [4,5]. Therefore, many interface performance tests were carried out. In these tests, the water diffusion test is an effective method to evaluate the interface performance of composite insulators [6,7].

The 100 h water diffusion test, as specified in IEC 61109-1992 and IEC 62217-2012, can be used to evaluate the hydrolysis resistance of samples with and without sheath [8,9,10]. According to the standards, during the test, the current flowing through the samples should not exceed 100 μA. Since the composite insulator is composed of an outer layer and an inner core rod, the currents measured by the double-electrode structure specified in the standard include the currents of the outer sheath, the inner core rod, and the interface. The currents of the different components may increase under certain conditions. For example, the surface current will increase when the outer sheath is stained or aged. Moreover, the inner current increases when the interface performance is degraded or the core rod is defective. Therefore, it is difficult to locate the defects in the insulator [11]. To solve this problem, an improved current sensor to measure the currents at different positions is proposed in this paper. In this new current sensor electrode structure, the upper electrode is identical to that specified in the standard, and the lower electrode is composed of three concentric ring electrodes. The outermost, middle, and inner ring electrodes served to measure the currents of the outer sheath, the interface, and the inner core rod, respectively. In this study, water diffusion tests on composite insulators were carried out. Based on the test results, the influence of surface current on the ageing sheath was analyzed. In addition, insulator samples with different interfacial performances were tested. Furthermore, the value and phase of current were proven to be two effective indicators for locating defects. The new current sensor proposed in this paper was proven to be effective according to the test results.

## 2. Current Sensors and Specimens

### 2.1. Conventional Current Sensor Used in Water Diffusion Tests

**Figure 1:**
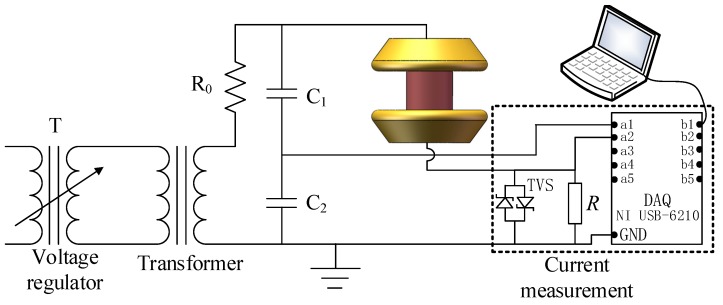
Test circuit structure.

The water diffusion test is an important method to evaluate the hydrolysis resistance of a short sample of a composite insulator. To carry out the water diffusion test, samples were cut from composite insulators as specified in [9]. Before boiling, the sample surface was cleaned with isopropyl alcohol and dried with filter paper. Then, the samples were boiled in a container for 100 h in deionized water with 0.1% by weight of NaCl. The conductivity of this solution is about 1750 ± 80 μS/cm at 20 °C. After boiling, the samples were immediately taken out of the boiling container and placed into another glass container filled with tap water at 20 °C for 20 min. Finally, the voltage test was performed in 2 h after the water diffusion test. The test device and current sensor are shown in Figure 1. In the voltage test, the voltage was increased at approximately 1 kV/s up to 12 kV, kept constant at 12 kV for 1 min, and then decreased to zero.

During the voltage test, the sample was placed between the parallel plate electrodes, as indicated in Figure 1. The power was supplied by an AC transformer which works at a frequency of 50 Hz. The maximum working voltage of the AC transformer is thus 50 kV. The voltage applied between the upper and lower electrodes was obtained by a capacitive voltage divider and the currents were measured by current measurement system. For the current measurement system, the current flowing through the sample is calculated via the equation below:

(1) I=U/R

where *I* (A) is the current flowing through the sample. *U* (V) is the voltage applied on the sampling resistor *R* (Ω). *R* is a sampling resistor with a resistance value of 1 kΩ. To protect the DAQ from overvoltage, a transient voltage suppressor (TVS) diode with a clamping voltage of 5 V was placed in parallel with the sampling resistor. When the voltage applied on the sampling resistor is higher than 5 kV, the TVS diode junction cascades, providing a low-impedance path for the current and protecting the DAQ. Therefore, the maximum current that can pass through the sampling resistor is 5 mA. The current signals were sampled by data acquisition (DAQ) at 20 ksamples/s. The DAQ is a NI USB-6210 (National Instruments Corporation, Austin, TX, USA). The measured current had an accuracy of ±1 μA.

### 2.2. Improved Current Sensor

**Figure 2:**
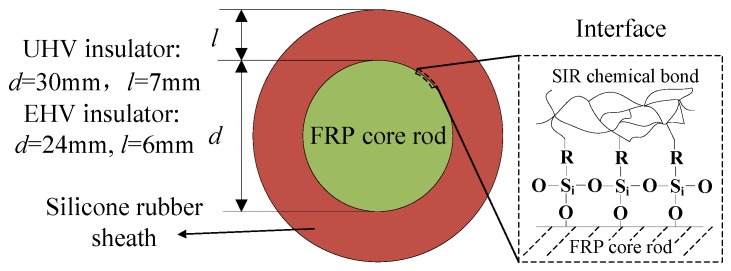
Structure of short sample.

A composite insulator is composed of a silicone rubber sheath and a FRP core rod. The sheath and core rod are bonded together by a coupling agent. A good quality interface is well bonded with Si-O-C chemical bonds. The structures of the short sample and the interface are shown in Figure 2. 

**Figure 3:**
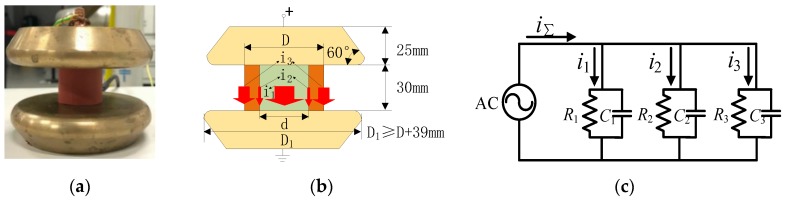
Principle of the traditional current sensor: (**a**) Conventional electrode; (**b**) Conventional electrode model; (**c**) Equivalent lumped circuit.

The conventional current sensor structure for the voltage test after the water diffusion test is shown in Figure 3 [12]. As depicted by Figure 3, current will flow through the sheath, interface and core rod when high voltage is applied on the sample.

In Figure 3b, i_1_, i_2_ and i_3_ are the currents flowing through the core rod, the interface and the outer sheath, respectively. The equivalent circuit is displayed in Figure 3c. In this circuit, three components of the sample can be expressed by lumped circuit parameters. *R*_1_ and *C*_1_ are the equivalent resistor and capacitance of the core rod component. *R*_2_ and *C*_2_ are the equivalent resistor and capacitance of the interfacial component, and *R*_3_ and *C*_3_ are the equivalent resistor and capacitance of the outer sheath component, respectively. All of them are the lumped values of the spurious parameters. Then, the currents and phases can be calculated as follows:

(2) $$I_{i}=U\cdot (\frac{1}{R_{i}}+j\omega C_{i})$$

(3) $$I_{\Sigma }=\sum_{i=1}^{3}I_{i}=\sum_{i=1}^{3}U\cdot (\frac{1}{R_{i}}+j\omega C_{i})$$

(4) $$\varphi_{i}=\arctan(\omega C_{i}R_{i})$$

where *I_i_* (A) is the current of the outer sheath, the inner core rod, or the interface. *U* (V) and *ω* (rad/s) are values of voltage and frequency respectively. I_Σ_ (A) is the total current flowing through the sample, and *φ_i_* (rad), *R_i_* (Ω), and *C_i_* (F) refer to the current-voltage phase difference, resistor, and capacity of each component, respectively. If one component is defective, the salt water will infiltrate into it during the boiling period, leading to a decrease in *R*_i_ (Ω) and an increase in *C_i_* (F). According to Equation (1), the value of *I_i_* (A) will therefore increase. 

**Figure 4:**
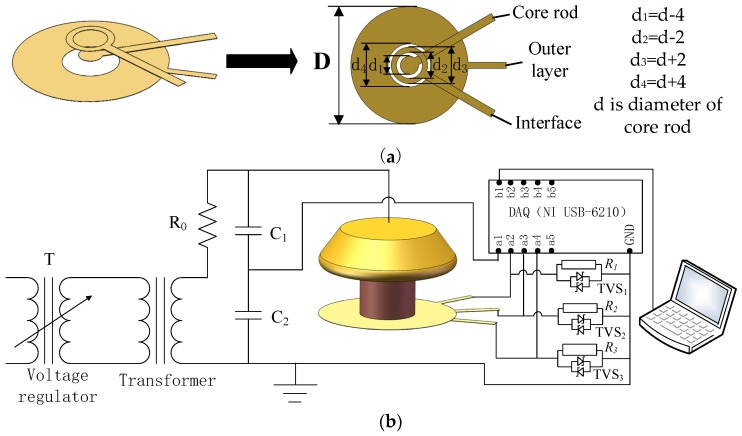
Improved current sensor: (**a**) The new lower electrode; (**b**) The new test circuit structure.

As depicted in Figure 3, the bottom surface of sample is in contact with the single lower electrode. Currents flowing through outer layer, interface, and core rod all reach the single lower electrode. Considering that any part of the three parts (outer layer, interface, or core rod) of the sample may be defective, currents need to be measured individually. However, the current measured by the traditional current sensor is the sum of three currents, therefore, the conventional current sensor cannot distinguish these currents from each other. To measure the current of each component in sample, the lower electrode in Figure 3 was redesigned as indicated in Figure 4a. The new test circuit structure is shown in Figure 4b.

As shown in Figure 4a, to the measure currents of the three components, three concentric ring electrodes were used as lower electrode. The parameter *d* is the diameter of the core rod as shown in Figure 3. To differentiate each part of the currents, an interval of 1 mm between adjacent ring electrodes is set in the new lower electrode. Because the aging occurs mainly on the surface layer of outer layer [13,14], the outer ring electrode is designed to be able to cover the whole surface layer. For the middle ring electrode, its radial width is 2 mm as shown in Figure 4a. The middle ring electrode is able to record the whole interfacial current because the width of the interface is far less than 1 mm [12]. As the diameter of the inner circular electrode is slightly less than the diameter of the core rod, the current flowing through the inner electrode can also be recorded. The currents flowing into three concentric electrodes were recorded via sampling resistors *R*_1_, *R*_2_ and *R*_3_ respectively. Each resistor has a resistance value of 1 kΩ. By analyzing three measured current components, the total currents can be obtained. The electrodes were made by thin conductive brass foil, which can ensure the flatness of the electrode contact surface. To avoid mutual conduction between electrodes, insulating tape was used for insulation. 

### 2.3. Comparison of Two Currents Sensors via Simulation Calculation

**Figure 5:**
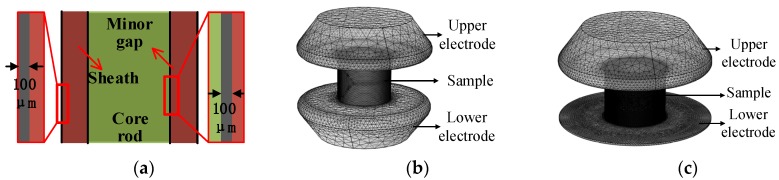
Simulation models: (**a**) Sample profile; (**b**) Conventional electrode; (**c**) Improved electrode.

To compare the conventional and improved current sensors, current flowing through the samples was simulated through finite element method. The simulation models are shown in Figure 5. Due to the poor adhesive performance of the interface in some bad quality samples, minor gaps may exist in the interface. Besides, the surface of silicone rubber sheath will be aged under the influence of the environment after operation, therefore, for ageing samples, the surface layer of silicone rubber can easily absorb water. Considering that the electrical conductivity of the sheath and interface may rise after being boiled, a thin layer of 100 μm was selected on the sheath surface and in the interface to simulate the minor defect, as shown in Figure 5a. 

The calculation of currents flowing through samples obey the equations below:

(5) $$J=(\sigma +j\omega \varepsilon_{0}\varepsilon_{r})\cdot E$$

(6) E=−∇V

where *J*(A/m^2^) is current density, *σ* (S/m) is the conductivity, ω (rad/s) is frequency of voltage, $\varepsilon_{0}$ (F/m) is the vacuum dielectric constant, $\varepsilon_{r}$ is the relative permittivity, *E* (V/m) is the electric field, and *V* (V) is the potential. 

Before the simulation, a voltage of 12 kV is applied on the upper electrode. As shown in Figure 1 and Figure 4b, the lower electrodes are in series with the sampling resistors which are grounded. To obtain the real and imaginary parts of the current, the simulation is solved in frequency domain. The conductivity and relative permittivity of materials are set as shown in Table 1.

**Figure 6:**
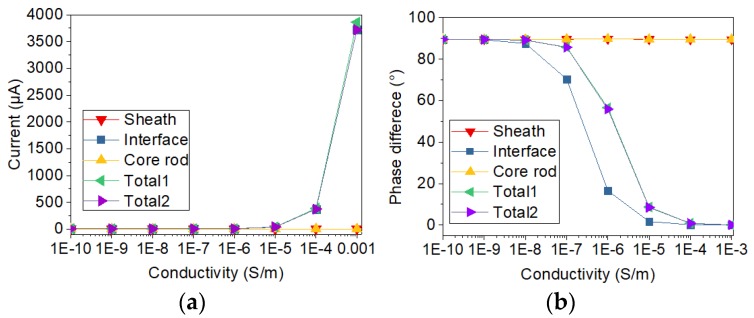
Current varying with conductivity of interface gap: (**a**) Change of current; (**b**) Change of phase difference.

**Figure 7:**
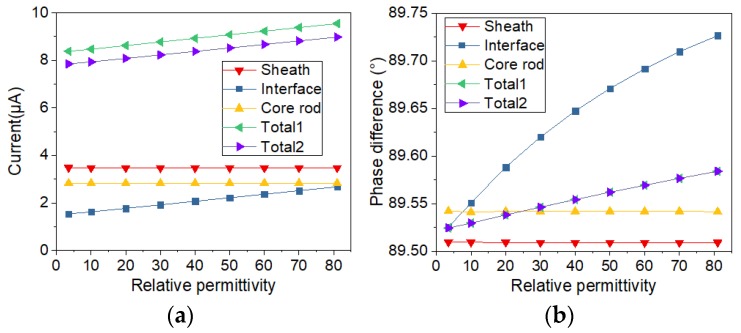
Current varying with relative permittivity of interface gap: (**a**) Change of current; (**b**) Change of phase difference.

**Figure 8:**
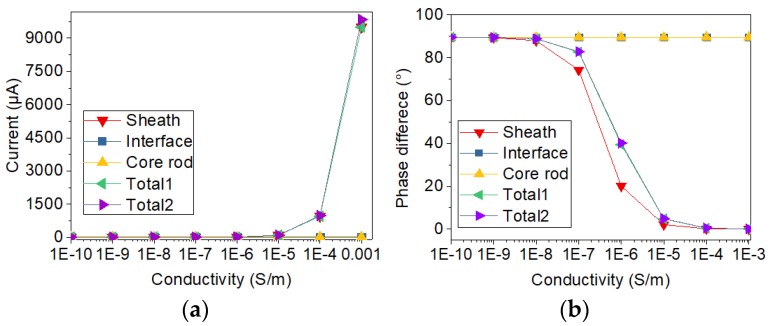
Current varying with conductivity of surface layer of sheath: (**a**) Change of current; (**b**) Change of phase difference.

**Figure 9:**
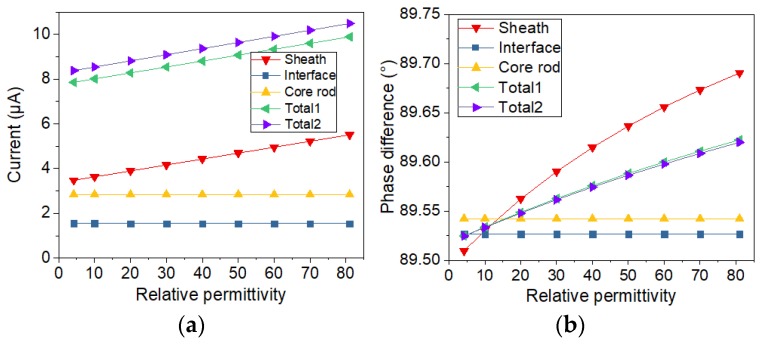
Current varying with relative permittivity of surface layer of sheath: (**a**) Change of current; (**b**) Change of phase difference.

In addition, in order to analyze the sample with and without defect, the conductivity of the thin layer shown in Figure 5a is changing from 10^−10^ S/m to 10^−3^ S/m. The relative permittivity of the thin layer is changing from 3.5 to 81. By calculating the currents with the two types of electrode structure, the variation of current with the change of electrical conductivity and relative permittivity was analyzed. Meanwhile, the two types of current sensors are compared. After the simulation calculation, the currents flowing through the electrodes are obtained by solving the surface integral of current on the electrode. Phase differences are calculated via subtracting current phase from voltage phase. The results of current and voltage-current phase difference are displayed in Figure 6, Figure 7, Figure 8 and Figure 9. 

In order to study the influence of a single factor, only one of two electrical parameters (conductivity and relative permittivity) is changed in a figure. The changed electrical parameters are shown in the titles of the figures. In Figure 6, Figure 7, Figure 8 and Figure 9, sheath, interface, and core rod in the legend represent the currents of different components simulated via the new current sensor. In Figure 6, Figure 7, Figure 8 and Figure 9, Total1 and Total2 are the total current tested via the new and conventional electrode structures. The current of Total1 is obtained by solving the sum of three concentric currents of new electrodes. The turrent of Total2 is obtained by solving the surface integral of current on the lower electrode. As depicted in Figure 6 and Figure 7, when the electrical parameters of sheath surface or interfacial gap change, the currents of sheath and core rod remain the same. However, as the conductivity of interfacial defect increases, the value of the interface current increases and the voltage-current phase difference of the interface current decreases. Besides, the change of currents and phase difference is small when the relative permittivity of minor gap rises from 3.5 to 81. Similarly, according to Figure 8 and Figure 9, when the conductivity and permittivity of a certain part (sheath surface) in the short sample change, the current of the certain part will change accordingly. Figure 6, Figure 7, Figure 8 and Figure 9 show that the total currents obtained via the two current sensors are almost the same. However, the currents of different parts changed differently when the electrical parameters of a certain part is changed. Compared with other parts, the current of the defective part is the largest and its phase difference is the smallest. By comparing the currents and phase differences among different parts, the improved current sensor is effective in locating the defects. Moreover, compared with the change of permittivity, the change of conductivity has greater influence on the results of current measurement. 

### 2.4. Analysis of Voltage Difference between Adjacent Ring Electrodes 

**Figure 10:**
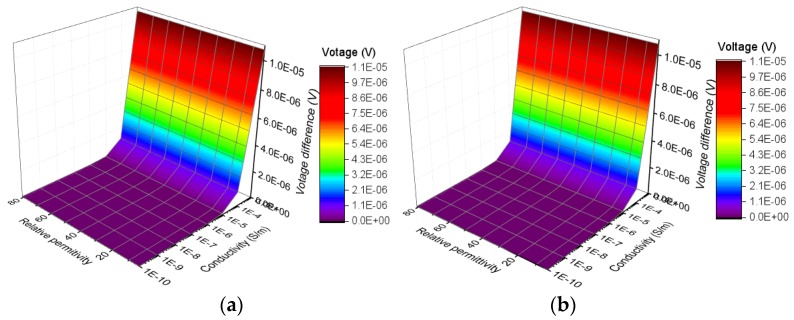
Change of voltage difference between adjacent electrodes with electrical parameters of interface: (**a**) Voltage difference between outer ring electrode and middle ring electrode; (**b**) Voltage difference between inner ring electrode and middle ring electrode.

**Figure 11:**
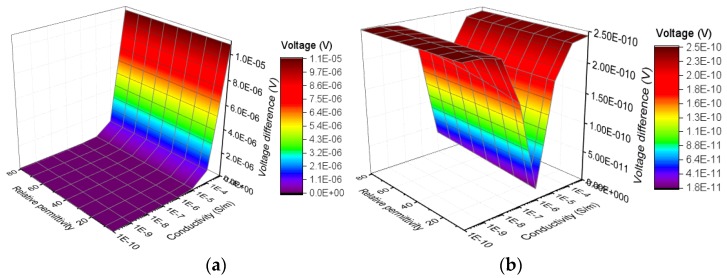
Change of voltage difference between adjacent electrodes with electrical parameters of surface layer of sheath: (**a**) Voltage difference between outer ring electrode and middle ring electrode; (**b**) Voltage difference between inner ring electrode and middle ring electrode.

Because a high voltage of AC 12kV is applied on the sample during the voltage test, a voltage difference between adjacent ring electrodes in bottom electrode may exist. For example, the voltage on the outer ring electrode may rise when the outer layer of sample is defective. Similarly, the voltage on the middle ring electrode may rise when the interface is defective. The voltage difference between adjacent ring electrodes may cause surface currents on the bottom of samples, which may lead to measurement errors. To analyze the influence of voltage differences between adjacent electrodes, the changes of voltage difference with electrical parameters of samples are extracted from the simulation results, which are shown in Figure 10 and Figure 11. 

The calculation of voltage difference follows the formula below:

(7) $$V_{ij}=\left|V_{i}-V_{j}\right|$$

where *V_ij_* (V) refers to voltage difference between the adjacent ring electrodes numbered *i* and *j*. *V_i_* (V) and *V_j_* (V) are the voltage of adjacent ring electrodes numbered *i* and *j*, respectively. As shown in Figure 10 and Figure 11, as the electrical parameters of silicone rubber sheath or interface change, the highest voltage difference between adjacent electrodes is 1.1 × 10^−5^ V. Because a spacing of 1 mm is set between the adjacent ring electrodes, the currents caused by the voltage difference is so small that can not cause obvious measurement error.

### 2.5. Test Specimens

The samples used in the test include new insulators and aged insulators, as listed in Table 2. During the manufacturing process of composite insulators, coupling agent coating and surface grinding of the core rod are two important procedures. When there are problems in the manufacturing process, minor gap may exist in certain part of interface due to improper handling. To study the effect of these two procedures on the interface performance, samples labelled with NN1~NN3, YN1~YN3, YY1~YY3, and NY1~NY3, as listed in Table 2, were tested. The samples from aged ultra-high voltage (UHV) composite insulators are labelled with the letters H, M, and L. The letters H, M, and L indicate that the samples are from the high, middle, and low voltage sides of the insulator. In addition, three samples from aged extra-high voltage (EHV) composite insulators, labelled S1~S3, were also investigated.

**Figure 12:**
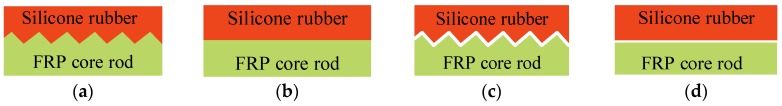
Interface of different performance caused by different production process: (**a**) YY1~YY3; (**b**) YN1~YN3; (**c**) NY1~NY3; (**d**) NN1~NN3.

A comparison of good and bad quality interfaces caused by the production process is shown in Figure 12.

As depicted in Figure 12, when the surface of core rod is well ground, the contact surface between the core rod and silicone rubber sheath is increased. Besides, according to the adhesive theory of the silane coupling agent [15,16,17], the coupling agent causes chemical bond cross-linking between the silicone rubber and the core rod. If the coupling agent is not well coated, the adhesion between silicone rubber and core rod will be weak. The two technologies help to enhance the bonding strength of the interface.

## 3. Current Analysis of Samples via the Two Current Sensors

### 3.1. Current Analysis of Insulator with Different Interfacial Properties 

**Figure 13:**
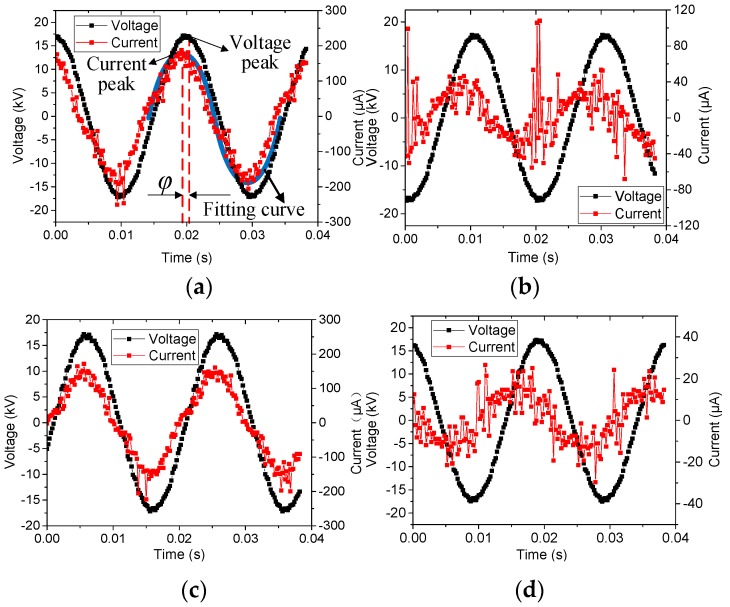
Voltage and current waveforms of sample L4 by the conventional and new electrode setups: (**a**) Total current; (**b**) Interface current; (**c**) Outer sheath current; (**d**) Core rod current.

After boiling, the currents flowing through the samples were measured by both the conventional and improved current sensors. Taking sample L4 as an example, the measured current and voltage waveforms are shown in Figure 13. Except for Figure 13a, which was measured by the conventional electrode setup, all other waveforms were obtained by the new electrode setup. As indicated in Figure 13, the voltage wave is close to ideal sinusoidal waveform. However, the current waveform has many burrs and is affected by background noise, such as corona discharge. The smaller the current amplitude is, the more obvious the burrs are. In Figure 13a, *φ* refers to the phase difference between the applied voltage and total current. 

To make a visual comparison of all samples from the new EHV composite insulators, the water diffusion test was carried out on these samples. Then, the original and new electrode setups were utilized to perform the voltage test. The measured results are shown in Figure 10.

**Figure 14:**
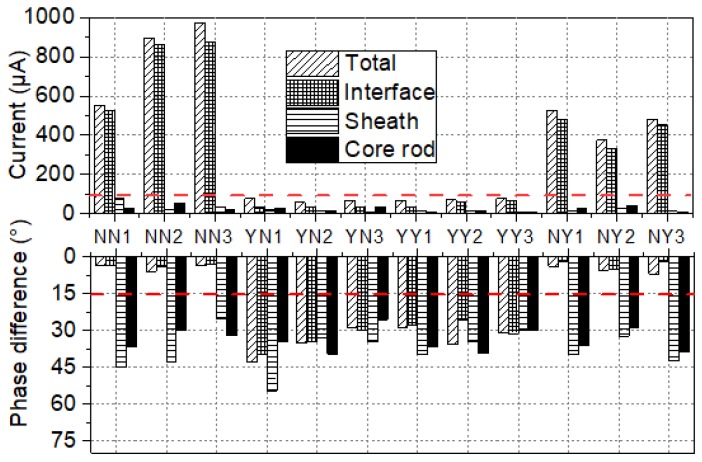
Current of four types of samples.

These samples were cut from new EHV composite insulators. Similarly, as shown in Figure 14, the currents of samples labelled with NN1~NN3 and NY1~NY3 are far beyond 100 μA in the voltage tests after 100 h water diffusion tests. Besides, the value of the interface current is close to the total current, which indicates that the interfacial performance is very poor. In addition, for the samples whose current are beyond 100 μA, the phase difference φ between applied voltage and total current, and phase difference between applied voltage and interface current are all less than 15°, which means that the resistive current is much higher than the capacitive current. 

**Figure 15:**
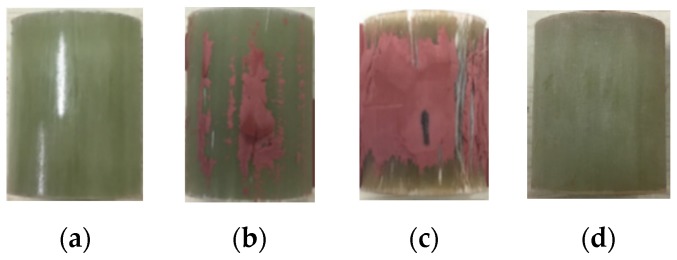
Core rod after stripping sheath: (**a**) NN1; (**b**) YY1; (**c**) YN1; (**d**) NY1.

The test results of the samples with four different interface-manufacturing processes indicate that the coupling agent coating on core rod is more essential in the production process. To observe the adhesion of interfaces visually, the silicone rubber is torn off from the core rod, as shown in Figure 15.

**Figure 16:**
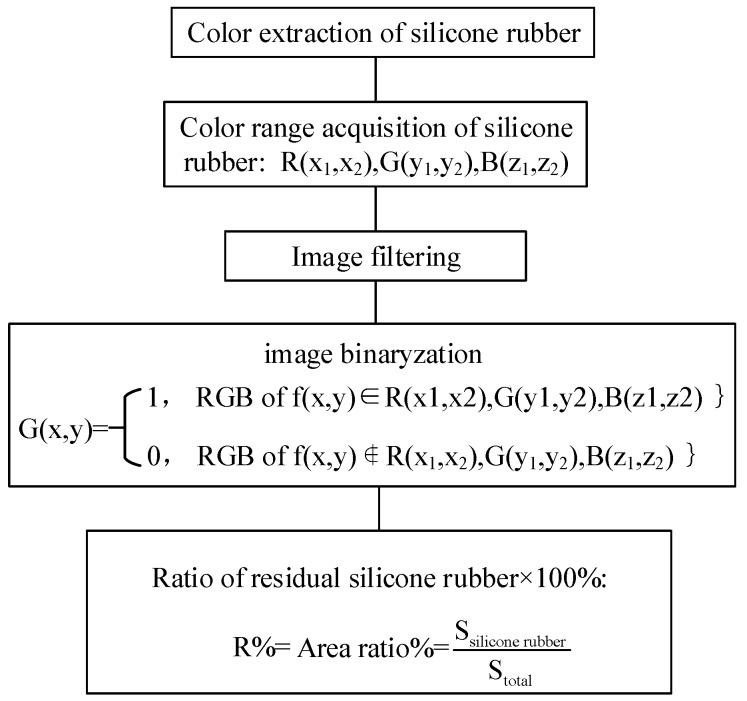
Image binarization process.

It is clearly shown that very little silicone rubber remained on core rod surface of samples labelled with NN1 and NY1. By means of color characteristics, the binarized image and ratio of residual silicone rubber can be obtained. The process of generating the binarized images is shown in Figure 16.

**Figure 17:**
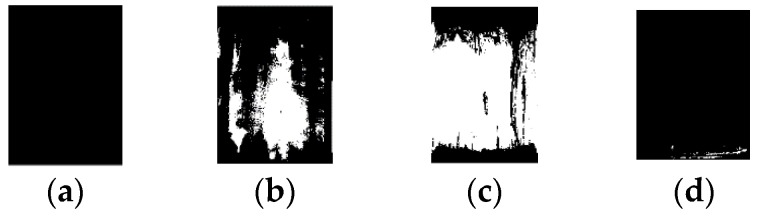
Binarized image of core rod after stripping sheath: (**a**) NN1; (**b**) YY1; (**c**) YN1; (**d**) NY1.

The binarized image is shown in Figure 17. As indicated in Figure 17, the white area represents the silicone rubber residue while the black area represents the core rod. In Figure 17, the area ratios of the residual silicone rubber of four samples are 0%, 25.6%, 64.0%, and 0.6%, respectively.

Similarly, the adhesion property of other samples coincides with this phenomenon. The area ratios of the residual silicone rubber of the other samples are shown in Table 3.

As the area ratios of silicone rubber residue of YY1~YY3 and YN1~YN3 are much higher than that of NN1~NN3 and NY1~NY3, it can be concluded their bonding performance is superior to the latter. 

### 3.2. Current Analysis of Aged UHV Large Tonnage Insulator 

**Figure 18:**
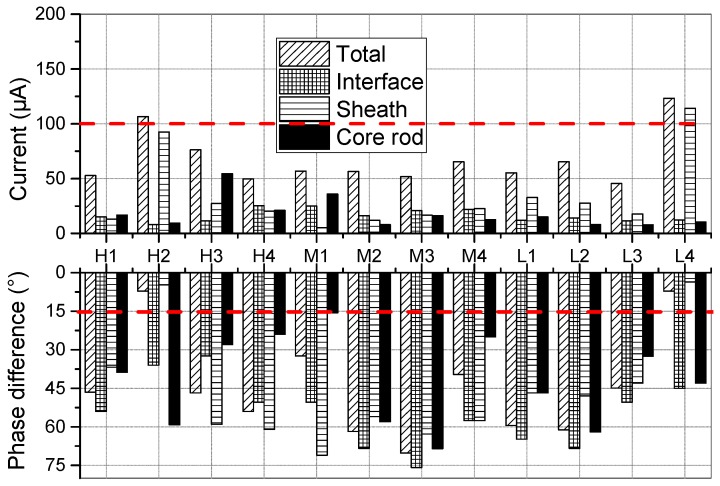
Results of the voltage test after 100h water diffusion test.

To analyze the performance of insulator after operation, the 100 h water diffusion test was carried out on samples from aged UHV insulators. Then currents of these samples were tested via two current sensors. The results are shown in Figure 18.

As indicated in Figure 18, the total currents of H2 and L4 exceed 100 μA, which does not meet the requirements in the standards [9]. For H2 and L4, the outer layer current takes a large proportion higher than 85% while the percentages of the interfacial current and core rod current are less than 10%. Thus, the total increase in current is mainly caused by the increase in the outer current. At the same time, it is worth noting that the phase differences φ between the applied voltage and the total current of H2 and L4 are 7.2° and 7.5°, respectively, which implies that the resistive current accounts for most of the total current. Similarly, the phase differences of the outer sheath currents of H2 and L4 are also less than 15°. However, the phase differences of the interfacial and core rod currents are higher than 30°. Hence, the rise of the total current of H2 and L4 is mainly attributed to the decrease in the resistivity of the outer sheath. 

By comparing the amplitude and phase differences of different current components (interface, outer sheath, core rod), the increase in total current can be ascribed to different reasons. For instance, the rise of the outer sheath current can cause an increase in total current of H2 and L4. Thus, the new current sensor can be used to analyze the location of defect by measuring the current components of each part.

#### 3.2.1. Analysis of the Reason for the Rise in the Outer Layer Current

The test results shown in Figure 18 indicate that the high value of the total currents of H2 and L4 after the water diffusion test is mainly due to the outer sheath current. To investigate further, the surface and internal silicone rubbers were cut from H2 and their microscopic appearances were observed by scanning electron microscopy (SEM).

**Figure 19:**
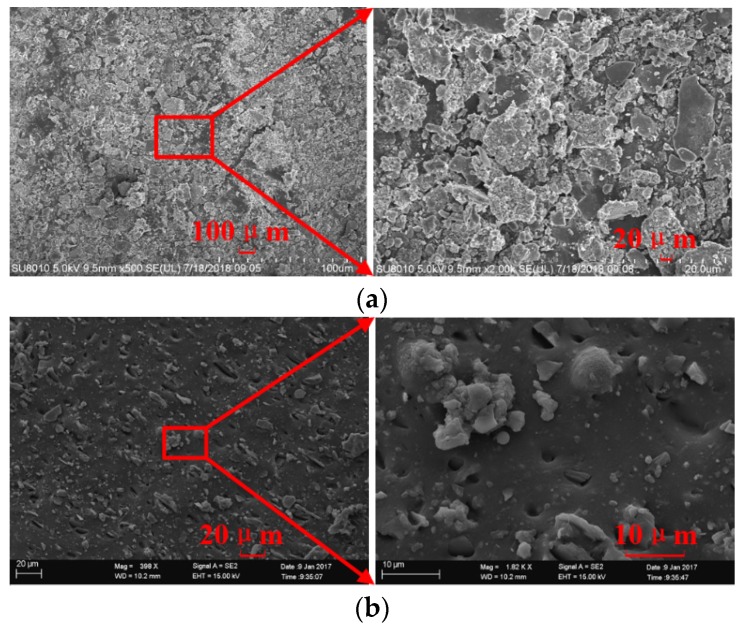
Microscopic appearance of silicone rubber: (**a**) Surface layer; (**b**) Inner layer.

**Figure 20:**
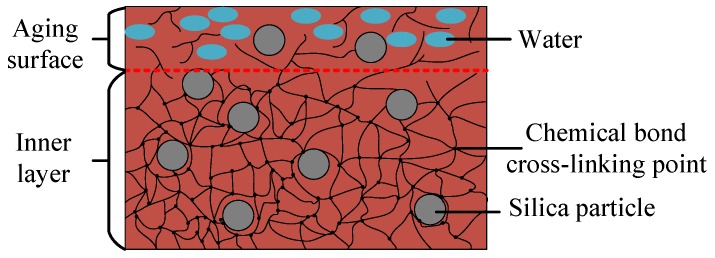
Water intrusion model.

The microscopic appearances of the surface and the inner silicone rubber are shown in Figure 19. The surface of silicone rubber is covered with layers of small particles, while the internal silicone rubber is much denser. After being subjected to high voltage, ultraviolet light, etc., for 9 years, the silicone rubber surface seems to be aged and cracked [18,19,20,21,22,23]. After boiling for a certain time, the moisture penetrated into the powdered layer of the silicone rubber surface, as illustrated in Figure 20.

Considering that the sample surface was cleaned with isopropyl alcohol and dried with filter paper before boiling, so contamination on surface has little influence on the outer layer electrical resistivity. So the moisture absorbed in surface layer is the main reason for the decrease of electrical resistivity of surface layer.

#### 3.2.2. Test after the Surface Layer of Silicone Rubber is Removed

**Figure 21:**
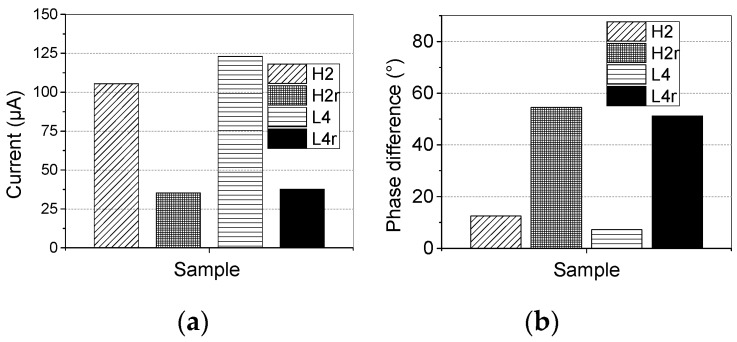
Comparison of the current and phase difference of samples H2 and L4 before and after the removal of the surface layer: (**a**) Current; (**b**) Phase difference.

To verify the previous analysis, a layer of silicone rubber surface of H2 and L4 were removed. The thickness of the removed layer was 1 mm. Then, the total current was tested again. The results before and after the removal of the aged composite insulator surface layer are presented and compared in Figure 21.

In Figure 21, H2r and L4r are the samples whose surface layers were removed. Obviously, after the surface layer was removed, the values of the total current are all below 40 μA. Meanwhile, the phase difference *φ* between applied voltage and total current are all increased from less than 15° to higher than 50°, which means a great reduction in the resistive current component. These results confirm that the increase in the outer layer current of H2 and L4 is caused by the ageing of the silicone rubber surface.

### 3.3. Current Analysis of Samples from the EHV Insulators after Operation

**Figure 22:**
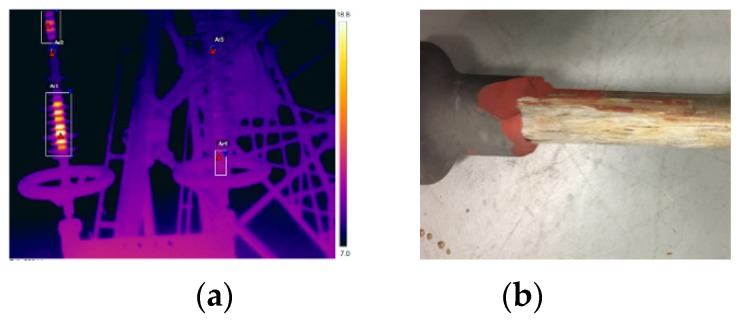
High temperature heating insulator: (**a**) Infrared image of the over-heated insulator; (**b**) Wood-like defect.

In South China, several composite insulators in service were found to be abnormally heated in 2017. This batch of products had been placed into service for only two years. Several positions of the insulators had temperature rises exceeding 10 K, which was a sign of severe defects. Thus, the defective insulators were subsequently replaced from the line. Meanwhile, some non-over-heated insulators were also removed for comparative analysis. After anatomical analysis, severe wood-like defects existed at the high voltage end of the abnormally heated insulators. The infrared image and the wood-like defects of the insulator core rod are displayed in Figure 22.

**Figure 23:**
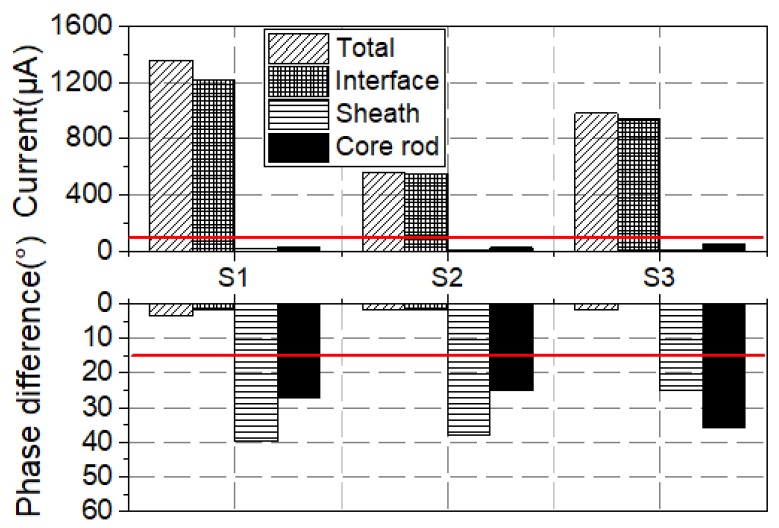
Measured results of voltage tests after water diffusion testing.

To analyze the interfacial property of this batch of products, samples named S1~S3, as shown in Table 2, were cut from the non-over-heated insulators that had been put into service for 2 years. Afterwards, the 100 h water diffusion tests were carried out. The measured results are presented in Figure 23. 

**Figure 24:**
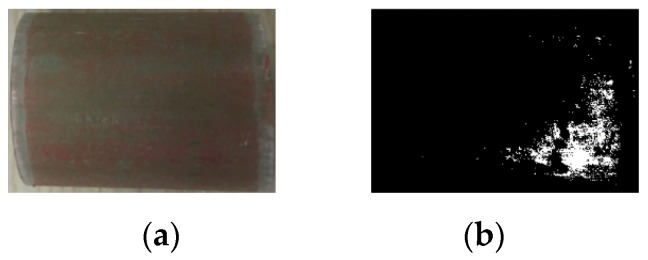
Anatomy observation: (**a**) Core rod without sheath; (**b**) Binarized image of the core rod.

The total currents and interface current components of the three samples all exceed 100 μA. In addition, all the phase differences between the applied voltage and the total current, and between the applied voltage and the interface current are less than 15°. Thus, the interface of the three samples was poorly bonded. To test the adhesion of the samples, the silicone rubber sheath was peeled off from the core rod of S1-S3. The residual silicone rubber on the core rod of S1 is shown in Figure 24a. and its binarized image is also displayed in Figure 24b. In Figure 24b, the residual silicone rubber area ratio is 5.4%. Besides, the residual silicone rubber area ratios of S2 and S3 are 4.2% and 3.9%, respectively. The results show a poor interface adhesion of this sample.

To further analyze the bonding performance of this batch of insulators, high voltage insulation test and dissection test of fifty-two samples were carried out. During the high voltage insulation test, abnormal heating was found in twelve samples, which means that the interfacial performance of these samples did not meet the standard. Besides, the interfacial defects were found in six samples during the dissection test. The high ratio of unqualified and defective samples show that the interfacial performance of this batch of samples was poor.

## 4. Discussion

**Figure 25:**
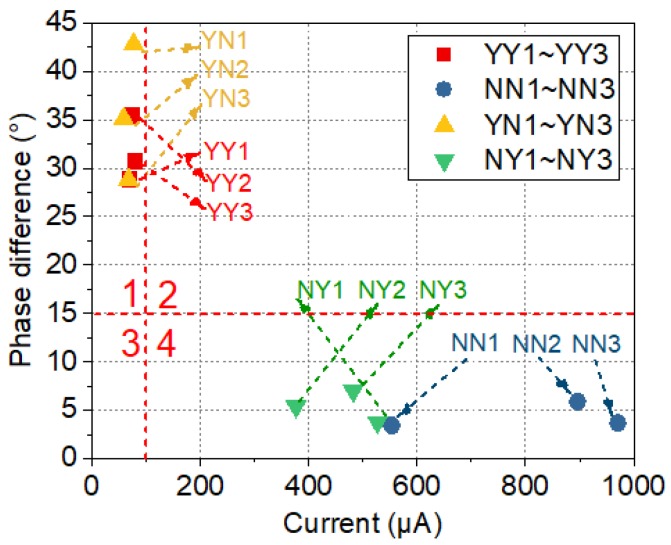
Two-dimensional scatter plot of the current and phase difference.

The above analysis indicates that the current and phase difference are closely related to defect information. Therefore, they are further analyzed. To determine the correspondence between the two parameters and the defects, this study analyzes the current and phase difference of the samples of NN1~NN3, and YY1~YY3, which are two samples with different typical interface properties. The results are plotted in Figure 25, which is divided into four regions by a current value of 100 μA and a phase difference of 15°. The definition of the four regions is listed in Table 4.

The state of the sample can be inferred by the region that the measured value falls in. The regions from 1 to 4 can be defined as normal, meaningless, pending and defective, respectively. In region 1, the current value is less than 100 μA and the phase difference is greater than 15°. If all pairs of values (current, phase difference) fall in this region, such as that of YY1~YY3 and YN1~YN3, then the sample can be regarded as free of defects. Due to the presence of defects such as poor adhesion, cracking in the core rod, or surface ageing, water easily penetrates into the defects. This will result in a reduction in resistance, and therefore, an increase in the current and a decrease in the phase difference. As long as one pair of values (current, phase difference) falls in region 4, where the current is higher than 100 μA and the phase difference is less than 15°, the sample is considered defective. Samples NN1~NN3 and NY1~NY3 are considered to have defects as the current values fall in region 4. By checking which component fall in region 4, the defect position can be further determined. Among all four regions, region 2 is meaningless as the phase difference is always lower than 15° when the current is above 100 μA. 

## 5. Conclusions

An improved current sensor was proposed based on the conventional current sensor. With the improved electrode structure, currents flowing through sheath, interface, and core rod of short sample can be measured separately. Based on the finite simulation of two types of electrodes, the new current sensor is proved to be effective in measuring the currents of different parts in short samples.

In the test of the samples from the new EHV composite insulators with four different interfacial properties, the improved electrode structure is proven to be effective in locating the defect in interface. Moreover, the area ratio of the residual silicone rubber also confirmed the bonding strength. In the water diffusion test of the aged UHV composite insulator, it can be observed that the current increase was caused by the outer current. Using SEM analysis, it was found that the water absorption on the surface layer of the silicone rubber was enhanced, resulting in an increase in the surface current. In addition, the test results of samples from the aged EHV composite insulators show that the interface performance of this batch of insulators was poor after two years of service. 

According to the four sub-regions divided by the current and voltage-current phase difference, the defect information can be judged according to the region in which the measured values fall. The production process of composite insulators is very complex and the insulators are sometimes operated under extremely harsh conditions, therefore, the defect may exist in any part of the composite insulators. As the improved current sensor can be used to locate defect of composite insulators, it is of significance in engineering practice.

## Figures and Tables

**Table 1:** Electrical parameter of material.

Component	Material	Conductivity (S/m)	Relative Permittivity
Silicone rubber sheath	Silicone rubber	1× 10^−14^	3.5
Core rod	Fiber reinforced plastic	1× 10^−14^	5.0

**Table 2:** Sample information.

Number	Coupling Agent	Core rod Grinding	Operation Years(a)
YY1~YY3	√	√	new
YN1~YN3	√	×	new
NY1~NY3	×	√	new
NN1~NN3	×	×	new
H1~H4	√	√	9
M1~M4	√	√	9
L1~L4	√	√	9
S1~S3	√	√	2

**Table 3:** Area ratios of the residual silicone rubber of eight samples.

Sample	NN2	NN3	YY2	YY3	YN2	YN3	NY2	NY3
Area ratios（%）	0.1	0.1	85.7	48.8	69.5	56.0	1.3	0.5

**Table 4:** Coordinate area division.

Region	Current Range	Phase Range
1	<100 μA	>15°
2	>100 μA	>15°
3	<100 μA	<15°
4	>100 μA	<15°
